# Ocean Acidification and Human Health

**DOI:** 10.3390/ijerph17124563

**Published:** 2020-06-24

**Authors:** Laura J. Falkenberg, Richard G.J. Bellerby, Sean D. Connell, Lora E. Fleming, Bruce Maycock, Bayden D. Russell, Francis J. Sullivan, Sam Dupont

**Affiliations:** 1 Simon F.S. Li Marine Laboratory, School of Life Sciences, The Chinese University of Hong Kong, Shatin, New Territories, Hong Kong SAR, China; 2 SKLEC-NIVA Centre for Marine and Coastal Research, State Key Laboratory for Estuarine and Coastal Research, East China Normal University, 500 Dongchuan Road, Shanghai 200241, China; richard.bellerby@niva.no; 3 Norwegian Institute for Water Research, Thormølensgate 53D, N-5007 Bergen, Norway; 4 Southern Seas Ecology Laboratories, The Environment Institute, School of Biological Sciences, The University of Adelaide, Adelaide, SA 5001, Australia; sean.connell@adelaide.edu.au; 5 European Centre for Environment and Human Health, University of Exeter Medical School c/o Knowledge Spa RCHT, Truro, Cornwall TR1 3HD, UK; l.e.fleming@exeter.ac.uk (L.E.F.); bmaycock@iinet.net.au (B.M.); 6 Swire Institute of Marine Science and School of Biological Sciences, The University of Hong Kong, Pokfulam Road, Pok Fu Lam, Hong Kong SAR, China; brussell@hku.hk; 7 Prostate Cancer Institute, Galway Clinic, Doughiska, H91HHT0 Galway, Ireland; frank.sullivan@galwayclinic.com; 8 Department for Biological and Environmental Sciences, University of Gothenburg, Kristineberg 566, 45178 Fiskebäckskil, Sweden; sam.dupont@bioenv.gu.se

**Keywords:** ocean acidification, global climate change, human health, seafood, malnutrition, air quality, respiratory health, biodiversity loss

## Abstract

The ocean provides resources key to human health and well-being, including food, oxygen, livelihoods, blue spaces, and medicines. The global threat to these resources posed by accelerating ocean acidification is becoming increasingly evident as the world’s oceans absorb carbon dioxide emissions. While ocean acidification was initially perceived as a threat only to the marine realm, here we argue that it is also an emerging human health issue. Specifically, we explore how ocean acidification affects the quantity and quality of resources key to human health and well-being in the context of: (1) malnutrition and poisoning, (2) respiratory issues, (3) mental health impacts, and (4) development of medical resources. We explore mitigation and adaptation management strategies that can be implemented to strengthen the capacity of acidifying oceans to continue providing human health benefits. Importantly, we emphasize that the cost of such actions will be dependent upon the socioeconomic context; specifically, costs will likely be greater for socioeconomically disadvantaged populations, exacerbating the current inequitable distribution of environmental and human health challenges. Given the scale of ocean acidification impacts on human health and well-being, recognizing and researching these complexities may allow the adaptation of management such that not only are the harms to human health reduced but the benefits enhanced.

## 1. Introduction

There is an intimate link between the health and well-being of humans and our biosphere, particularly the hydrosphere. Oceans provide nutrition, medications, mental and physical health benefits, climate control, mitigation of both CO_2_ and global warming, and coastal protection [1,2,3,4,5]. However, the provisioning of these resources can be altered to act as stressors of human health. That is, human health and well-being can be negatively affected by a range of biological changes, including modification of the timing, magnitude, and nutritional value of marine foodstuffs; exposure to toxins found in harmful algal blooms; exposure to viruses and antibiotic microbial resistant bacteria; drowning and injury secondary to extreme weather; or contact with wildlife such as jellyfish [6,7,8,9].

The interactions between humans, our health, and our oceans are likely to be increasingly modified as human-driven changes to the environment intensify. As negative changes in global oceans manifest as costs to human well-being, the health problems and environmental solutions are becoming increasingly recognized across national and international scales, attracting worldwide participation and continued global political focus in terms of the Sustainable Development Goals, UN Paris Climate Agreement, UN Decade of Ocean Science for Sustainable Development (2021–2030), and reports and publications by national and international organizations [10]. Implementation of policies and actions to counter these issues is, however, yet to be fully realized.

## 2. Ocean Acidification

The global threats from carbon dioxide (CO_2_) accumulating in the world’s oceans and driving ongoing ocean acidification are becoming increasingly evident. As humans extract and burn fossil fuels and drive deforestation, CO_2_ in the atmosphere increases, with levels now far exceeding those that occurred in preindustrial times [11,12]. Of the released CO_2_, around 25% is taken up by the oceans where it interacts with seawater and forms carbonic acid, leading to a reduction in pH, an increase in acidification, and an alteration of carbonate chemistry [13,14,15]. The average surface pH of the ocean has already decreased by 0.1 unit since the beginning of the Industrial Revolution, and a further 0.3–0.4 unit decrease is expected by the end of the century [13]. As pH is measured on the logarithmic scale, this corresponds to a doubling in acidity by 2100. These changes are happening at an unprecedented scale and speed, rapidly exposing marine ecosystems to conditions they have not experienced over millions of years.

To understand the future consequences of this ongoing CO_2_ uptake on marine ecosystems, modeling using several representative concentration pathways (RCPs) is often used. For example, the IPCC 2019 report used scenarios including RCP2.6 and RCP8.5, the former where society implements immediate change and the latter where we continue as we are (the “business as usual” scenario) [12]. As CO_2_ concentrations increase and acidification progresses, human health and well-being are set to be affected through a complex variety of routes, typically mediated by marine biota. The effects on biota are currently investigated using a range of observational, experimental, and modeling approaches that consider futures characterized by the CO_2_ levels predicted for the different RCP scenarios [16]. The vast literature of these studies over recent decades reveals ocean acidification is having a permeating and insidious influence on the oceans’ ecosystems and that the resulting changes are ubiquitous.

A wide range of organisms, from bacteria to fish, have been demonstrated to be negatively impacted by ocean acidification. As acidification progresses, many organisms must invest additional energy to maintain their acid-base balance, metabolic processes, or other biological functions, with consequences for their growth, reproduction, and survival. Consequently, around 50% of all the species tested in the laboratory have shown negative responses to ocean acidification [17]. On the other hand, some photosynthetic organisms can benefit from the increased concentration of CO_2_ where it is used for photosynthesis [18,19]. Such changes at the organism level can modify the balance between species and drive profound ecosystem changes that influence human health and well-being [17]. Negative impacts of ocean acidification are already visible in some parts of the world; for example, the observed collapse of the oyster aquaculture industry along the west coast of the United States in 2007 has been attributed to ocean acidification [20]. Such impacts, and consequences on human health and well-being, are anticipated to intensify with future acidification.

The study of ocean acidification effects on human health and well-being involves understanding substantial complexity, making this particular driver one of the more challenging to comprehend and manage. Compared to studies of other stressors, which tend to focus on direct effects (extreme events such as floods or storms, enrichment of chemical additives, or persistent elevated temperature; e.g., [21]), ocean acidification manifests in layers of complexity involving indirect effects and interactions [22,23]. While studies of ocean acidification initially focused on the potential direct effects (e.g., the effects of low pH on calcifying organisms [24]), research now incorporates the ecosystem-level complexity of indirect effects (e.g., [25]). Effects of CO_2_ are understood to propagate through food webs [25], to modify the availability and nutritional value of primary producers (e.g., seaweeds [26]) and their consumers (e.g., herbivores [27]), as well as their toxicity to humans [28]. As such, we highlight here that ocean acidification is very much an emerging human health issue of substantially greater complexity, and possibly scale, than currently appreciated.

Human health and well-being is an emerging area of study for ocean acidification research, and the benefits of managing its impacts in this context will be substantial. Many people are routinely exposed to the effects of ocean acidification via the consumption of seafood, breathing the air around them, and their physical and mental experiences of marine ecosystems. Other, less easily recognizable effects include those linked to the occurrence of biodiversity and, therefore, opportunities to develop new medicines. This exposure can influence human health in complex, and interacting, ways. Where these effects materialize, we need to recognize which aspects of the social-ecological systems [29] can be maintained or adapted to protect human health and well-being.

## 3. The Anticipated Influences of Ocean Acidification on Human Health

Here, we examine the ways in which ocean acidification could have impacts on human health in terms of four pathways: (1) malnutrition and poisoning via altered food quantity and quality, (2) respiratory issues via impaired air quality, (3) mental health impacts via modification of natural spaces, and (4) decreased opportunity to develop and obtain medical resources via loss of biodiversity. For our initial discussion of the human health impacts, we explore each of these pathways separately (as summarized in Table 1) and then synthesize the discussion, highlighting links and interconnectedness among the different pathways.

### 3.1. Pathway 1—Malnutrition and Poisoning Via Altered Food Quantity and Quality

#### 3.1.1. Quantity and Nutritional Composition of Seafood

Globally and historically, a key resource provided by the oceans is food obtained from fisheries and aquaculture. Of the global population of over 7.8 billion people, more than 4.5 billion obtain at least 15% of their animal protein intake from fish [30]. In some countries, the share of protein from fish can be greater than 50%, including in West Africa; Asian coastal countries; and many small island states (e.g., Gambia, Sierra Leone, Ghana, Cambodia, Bangladesh, Indonesia, Sri Lanka, and the Maldives) [51]. It is important to note that such patterns of dependence on ocean-based food security influence where the most vulnerable communities are found; 14 of the 50 most vulnerable nations are found in the Asia Pacific region, including smaller island nations such as Kiribati, Tonga, and the Cook Islands, as well as highly populated countries including China, Indonesia, Thailand, and North Korea [52]. Currently, 149 million children under five years of age are stunted, with 68% of these children found in Asia. Moreover, 55% of all malnourished children are found in Asia [53]. Of the 5.3 million children that die from preventable causes, nutrition-related factors contribute to 45% of the deaths [53,54]. While many of the currently observed deaths are typically related to malnutrition resulting from poverty rather than environmental impacts on the food chain, this statistic highlights the importance of the quantity and quality of the nutritional composition of food in human health (i.e., the avoidance of stunting and wasting).

Ocean acidification and its chemical changes can have direct effects on the physiology of consumed marine species at key life history stages (e.g., eggs, larvae, juveniles, and adults), such that their survival and, therefore, availability, is altered. For commercially important stocks of fish, particularly wild-capture fisheries, concern centers on population-level processes that are disrupted by ocean acidification, such as recruitment (i.e., the number of fish surviving to enter the fishery). Consequently, early life history stages have been a strong research focus. In the context of fish, studies have found negative effects of increased CO_2_ on the survival of eggs (as indicated by hatching success) and early larval stages for some species (e.g., summer flounder, *Paralichthys dentatus* [55] and Atlantic cod, *Gadus morhua* L. [56]). In contrast, some studies considering different fish species have not identified any effects of elevated CO_2_ on survival (e.g., orange clownfish, *Amphiprion percula* [57] and yellowtail kingfish, *Seriola lalandi* [58]). Similarly, reproductive processes have also been a focus; meta-analyses tend to indicate negative effects of ocean acidification on survival, growth, development, and reproduction across the different groups of organisms considered (e.g., fish, molluscs, echinoderms, and crustaceans) [59,60]. Where focus has been placed on cultured species, the effects have been found to be more diverse. The culture of shellfish in coastal waters is already experiencing decline in some regions because of the reduced carbonate saturation and mortality of juveniles unable to grow shells (e.g., oysters of the United States west coast; [32,61]). However, many aquaculture species and systems are thought to be less susceptible to ocean acidification because of the ability to monitor seawater conditions and implement mitigation strategies, particularly in land-based or recirculating systems that are commonly used for organisms such as scallops and prawns [33]. Further, many finfish aquaculture systems operate at high stocking densities that contain metabolic CO_2_ at extremely high concentrations [62]. While these species do show adverse effects of ocean acidification (e.g., pink salmon and Atlantic salmon [63,64]), the effects only manifest at CO_2_ concentrations greater than predicted to occur in the oceans by the end of the century [62]. Therefore, the fish produced in intensive aquaculture are either physiologically able to cope with ocean acidification or have been bred to optimize production under such conditions.

In addition to the direct effects on individual species, availability can also be modified by the indirect effects of ocean acidification. Such indirect effects are those where the species’ physiology is not affected but, rather, there is an effect on another species upon which the first depends for food, habitat, shelter, etc. For wild catch fisheries, changing habitats as a consequence of ocean acidification, such as degradation or loss of coral reefs or macroalgal forests [65,66], will induce ecosystem effects that alter the characteristics of dependent fisheries including, for example, salmon and Pacific halibut [31]. This altered species occurrence would result in changes to the composition of human harvest and, thus, diet.

Indirect effects can also be driven by changes in food source. In this context, phytoplankton are important as they form the foundation of the marine food web. A recent meta-analysis identified a range of phytoplankton responses to ocean acidification. Such differences can change the competitive fitness among types to affect community structure [67]. Where an individual species or the overall community structure is affected, this could modify interactions with other ecosystem components important to human health and well-being. For example, ocean acidification has been found to decrease phytoplankton polyunsaturated fatty acid (PUFA) concentrations, which has been associated with a reduction in copepods and negative effects on their somatic growth [68] and egg production [69]. It is worth noting that other studies have found no, or even positive, effects on plankton PUFA concentrations [70], and increases in zooplankton biomasses with these changes are likely driven by alterations in the phytoplankton community structure [71].

Facilitative ecological interactions could also be modified under ocean acidification resulting in the occurrence of species shifts and altered availability. That is, many fish and invertebrates rely on structures such as coral reefs for ecosystem services, including the provisioning of habitat and feeding grounds [72]. The persistence of these dependent species will likely be threatened under ocean acidification as coral reefs bleach and transition to more simple habitats [65]. Tropical fisheries will therefore likely be degraded by ocean acidification, with estimates of up to a 92% reduction in coral habitat, which would have a profound impact on those human populations dependent upon fisheries, particularly those that have seafood insecurity, such as small island developing states [73,74]. Similarly, ocean acidification and increased sea surface temperatures could combine to reduce the occurrence of giant kelp, a key habitat forming algae, which would have subsequent impacts upon both fisheries and aquaculture [75].

Ocean acidification has the potential to compromise the nutritional qualities of the seafood that is available, particularly in terms of traits such as lipids and proteins. For example, ocean acidification led to a reduction of lipids and proteins in a cultured whelk species [27]. A reduction in lipids is particularly concerning from a human health perspective given that polyunsaturated fatty acids (PUFAs)—especially, omega-3 PUFAs—are beneficial to ongoing human health and longevity; they provide fatty acids, carry fat-soluble vitamins, reduce the risk of heart disease, have anti-inflammatory properties, and aid in suppressing abnormal heart beats and promoting efficient blood circulation [76,77]. The benefits of PUFAs can be variable [78] and likely depend upon the source (i.e., supplements or fish), treatment period, dose, sample size, background intake level, patient history, and concurrent use of modern pharmacotherapy for cardiovascular disease prevention [78,79]. At this time, evidence supports the consumption of fatty fish, with omega-3 PUFA supplementation an alternative for those who do not consume fish. While both options are likely beneficial, fish is the preferred source of omega-3 PUFAs as it also provides additional, often underconsumed, nutrients [79]. Consequently, the loss of PUFAs under ocean acidification scenarios would have negative consequences for human health.

Ocean acidification could also have indirect effects on nutrition by modifying species interactions. In the context of trophic links, while some seafood species may not be directly affected, their food source may be modified, leading to a change in the species that are harvested for consumption. For example, diatoms cultured under ocean acidification conditions produced a ratio of long-chain polyunsaturated fatty acids to saturated fatty acids that was three times lower than was produced under contemporary conditions. This shift translated to the copepods that consumed the diatoms via trophic transfer, constraining their growth and reproduction [34]. Moreover, omega-3 long-chain PUFAs are known to progressively accumulate in aquatic food chains, meaning that reductions in production at lower levels could impact higher-level organisms and their consumers [80]. Consequently, ocean acidification may impact continued human access to these nutritional resources, as the higher trophic-level species that we rely on for sustenance are likely to reduce in nutritional value. Importantly, this shift will be irrespective of whether the seafood is sourced from wild fisheries or aquaculture, meaning that we are unlikely to be able to compensate through modified production technologies.

These biological changes are anticipated to combine to lead to reduced protein input, depleted food quality, and exacerbated human malnutrition particularly for vulnerable coastal and island communities in lower and middle-income countries [81,82,83]. Reduced availability will lead to changes to the accessibility of traditional fisheries and fishing grounds, obligating longer passage times and the acquisition of different fishing gears. Even where such changes are successful, there are likely to be increases in the price of fish, leading to encouragement of alternative food sources. While some consumers may try to obtain other similar products, ocean acidification will impact entire classes of fisheries, making it harder for people to source these products. In some countries or regions, this will mean there are fewer or even no alternatives available [84], while other regions will have alternatives that are less expensive but likely unhealthy and processed with more added artificial colors, flavors, and preservatives [85]. Such inability to access suitable alternative food sources will be detrimental where food quantity and nutritional value is affected, as well as where food safety is impaired.

#### 3.1.2. Chemical Contamination (Pollutants)

While we rely on the oceans to provide seafood, not all seafood is safe for human consumption due to pollution that results in enhanced chemical concentrations. Many chemicals added directly and indirectly to natural systems by human activities can be taken up by organisms and then transferred through the food chain, accumulating in tissues of organisms at higher trophic levels, including humans, and impacting their physiology [35]. Substances of concern include those that have long been recognized, such as heavy metals (mercury, aluminum, copper, iron, lead, arsenic, and zinc), as well as emerging toxicants such as pharmaceuticals (e.g., pain relievers, blood pressure modulators, cholesterol reducers, antidepressants, contraceptives, and antibiotics) and active ingredients in personal care products (e.g., detergents, perfumes, and sunscreens) [86]. These contaminants mainly enter the environment via municipal effluent discharges, particularly poorly treated sewage, with other sources including aquaculture, animal husbandry, and horticulture runoff and waste disposal [87]. Where chemicals occur in particularly high levels, they can lead to reduced human food safety [36]. A well-studied example of this includes the concentration of mercury in the muscle tissue of finfish and subsequent impacts on human neurologic and developmental health [88,89,90]. We also recognize that DDT is commonly concentrated in the liver, muscle, and adipose (fat) tissue of marine mammals, including whales and walruses [91,92], with exposure to this pollutant having the potential to influence cancer occurrence, neuropsychological dysfunction, and reproductive outcomes in humans [93,94].

The propagation of contaminants through the marine environment can be affected by ocean acidification, which has been observed to change the bioavailability of pollutants and intensify exposure and bioaccumulation, as well as enhance effects across biological levels of organisms from genes to ecosystems [36]. For example, mercury and some metals (e.g., aluminum, iron, zinc, copper, and lead) are often more bioavailable in acidified aquatic habitats [37,55,95]. Such increased availability appears to explain the enhanced cadmium accumulation in three marine bivalves exposed to ocean acidification (*Mytilus edulis*, *Tegillarca granosa*, and *Meretrix meretrix*; [96]). Alternatively, acidified seawater may enhance accumulation by causing epithelia damage, which results in easier cadmium penetration or impairs the capacity to exclude cadmium [96].

Changes in processes not directly related to chemical uptake may also modify the concentration of pollutants in consumed species. In terms of physiological processes, fish with higher growth rates tend to have lower mercury concentrations [97]. Therefore, it could be anticipated that fish whose growth rates are reduced under ocean acidification may exhibit increased mercury levels [36]. Human populations exposed through the consumption of seafood contaminated with mercury are subsequently at increased risk of neurologic and developmental health issues [89,90]. In terms of ecological processes, organisms are anticipated to be affected by changes to those at different trophic levels in food webs. For example, the amount of pollutants (including persistent organic pollutants, such as polychlorinated biphenyls (PCBs)) absorbed and amplified through the food web is anticipated to be linked with effect of environmental conditions on phytoplankton primary production ([36] and references therein). Where PCBs are accumulated in fish and consumed by humans, they are known to have neurological effects, particularly when transferred to fetuses across the placenta or to nursing infants via breastmilk [98].

#### 3.1.3. Redistribution and Accumulation of Natural Toxins

Poisoning from seafood, particularly shellfish, is a key pathway by which conditions in the oceans can negatively affect human health [39,99,100]. Shellfish can be harmful where they contain natural toxins that induce paralytic shellfish poisoning (PSP), neurotoxic shellfish poisoning (NSP), amnesic shellfish poisoning (ASP), or diarrheic shellfish poisoning (DSP). These toxins are accumulated from algae, often those that form harmful algal blooms (HABs) [101].

Ocean acidification can modify the abundance and chemical composition of harmful algal blooms in such a way that shellfish toxicity increases and, therefore, human health is negatively affected. Toxins, and algae which produce them, that have been found to be affected by ocean acidification include paralytic shellfish toxins (*Alexandrium tamarenese*) [39] and neurologic shellfish toxins, specifically brevetoxins (*Karenia brevis*) [99]. In both cases, ocean acidification has been found to lead to an increased algal growth rate [39,99], a change which could accelerate the production of toxic algae. Moreover, the toxicity of the blooms is likely to increase, although the way in which this could occur is likely to vary by HAB organism and toxin. For example, in *A. tamarenese*, the total cellular toxicity per cell is anticipated to increase due to an increase in cellular concentration of the more toxic derivatives, overwhelming the reduced toxins per cell [39]. In contrast, the higher growth rate of *K. brevis* would drive a higher total toxin production, despite the total brevetoxin production and profile remaining unchanged [99].

In addition to harmful microbial algae, bacteria can also cause illness via the release of toxins. For example, the bacteria *Clostridium botulinum* produces the very potent natural toxin botulin that, when ingested orally, blocks nerve functions, prompting respiratory and musculoskeletal paralysis [102]. Growth of this bacteria can be influenced by pH (although it is worth noting the pH treatment levels considered in this study are far lower than anticipated to result from near-term ocean acidification) [103].

### 3.2. Pathway 2—Respiratory Issues Via Impaired Air Quality

Human respiratory issues can be triggered by the aerosolization of natural toxins produced in harmful algal blooms. For example, blooms of the dinoflagellate *Karenia brevis* characterize “Florida red tides”. As *K. brevis* produces neurotoxins—specifically, brevetoxins—when the organisms are broken up in the surf, there can be the release of brevetoxin mixed into sea water aerosols [40]. Aerosol exposures during active Florida red tides have long been reported to result in upper and lower respiratory irritations in humans (e.g., [104]). Self-reported respiratory symptoms have been linked with inhaling brevetoxin aerosols at the time of red tide events [40,105]. Adverse respiratory effects are typically transient and self-resolving but can include upper airway irritation and discomfort, the exacerbation of asthma symptoms, and decreases in pulmonary functions lasting at least several days [6,40].

Ocean acidification can impact phytoplankton bloom dynamics. Where ocean acidification drives changes to the occurrence or composition of dinoflagellates—specifically, *Karenia brevis*—this could have implications for the occurrence of atmospheric brevetoxins and, therefore, human respiratory health. It has been found that future CO_2_ concentrations can lead to the enhanced growth of *K. brevis*, although no change in toxin production, indicating the two processes are not linked in this species [99]. Consequently, future ocean acidification could produce blooms with higher cell concentrations, increasing the risks for human health. 

### 3.3. Pathway 3—Mental Health Impacts Via the Modification of Natural Spaces

Mental health benefits derived from the oceans are largely associated with the aspects of livelihoods, recreational activities, and social connections. In terms of livelihoods, benefits can be derived from the economic independence associated with jobs provided by the oceans (e.g., tourism and the selling of fish). For example, approximately 300 million people find their livelihoods associated with marine fisheries, and 90% of those are in small-scale, artisanal fisheries [41]. The oceans also provide nature-based recreation and exercise opportunities (e.g., swimming, snorkeling, SCUBA diving, rock pooling, and coastal walking and running) that promote “nature-connectedness”, all of which have been shown to be a fundamental of mental health [42,43,106]. Importantly, it is habitats of high quality that are strongly associated with higher levels of nature connectedness [44]. Quantification of the social benefits of experiencing nature are also emerging; we recognize experiencing nature encourages physical activity and social interactions that lead to greater social cohesion, shared community values, trust, and a positive sense of community [47,48]. In satisfying our innate need to associate with the natural world (i.e., biophilia hypothesis [107]), enhanced physical, social, and psychological well-being are likely to be realized.

In the context of mental health impacts associated with livelihoods, ocean acidification is anticipated to result in decreased availability and nutritional value of fished species while also increasing chemicals and toxins of concern (discussed above), meaning less will be available for extraction from future oceans. The loss of availability and value of fish stocks have historically caused the collapse of regional fishing industries (e.g., in Europe), causing unemployment, financial pressures, societal stress, and a decline in the mental health of individual fishers and the fishing community.

The loss of habitats, or the loss of access to habitats, anticipated to result due to ocean acidification reduces ecosystem services, including recreation opportunities and the formation of associated social connections. That is, habitats valued by people are predicted to be degraded, including coral reefs, kelp forests, and seagrass meadows [45]. The sense of connection to nature that contributes to positive well-being in “blue spaces” may be further eroded by the more frequent occurrence of undesirable species (e.g., red tides and *Ulva* spp. infestations). Access to marine environments, and the habitats that are able to persist despite acidification, can also be limited through the migration of poisonous marine organisms. This migration is already being witnessed in response to climate change driven indirect effects. For instance, key foundation species in decline under ocean acidification and topicalization of temperate coasts have been replaced by the movement of poisonous organisms around the Iberian Peninsula (e.g., [46]) and the proliferation of Irukandji and box jellyfish along the North Australian coast [108]. These organisms are entraining into regions where coastal societies are not familiar with the dangers and are often ill-prepared to handle the increasing medical challenges, leading to fear and avoidance of oceans and coastal areas. Where citizens experience the declining services offered to coastal cultures (e.g., clean and productive oceans), the psychological feedback can be negative. Where present, perceived or manipulated higher biodiversity is related to greater positive perceptions, feelings of well-being, perceived stress reduction, interest, and intention to visit [109,110,111]. In contrast, where citizens experience a decline in the benefits of the coastal environment, they may form negative perceptions about visiting these areas. Such a loss of access to valued blue spaces would not only limit social interactions and connection to these outdoor spaces but also drive a further shift to indoor lifestyles. People who have reduced connections with nature are generally less motivated to protect nature [112,113]. Over time, such lifestyle alterations may lead to an intergenerational disconnect from nature that has long-term, pervasive impacts on human well-being.

### 3.4. Pathway 4—Decreased Opportunity to Develop and Obtain Medical Resources Via the Loss of Biodiversity

The vast biodiversity of the oceans offers a key opportunity for scientists looking to find and produce medicines and other natural products to counter the impacts of environmental stressors on human health. Of the 34,000 molecules of medicines or cosmetic interest in existence today, only 10 have been developed from ocean-based organisms [50]. Consequently, there are still opportunities for prospecting and extracting new compounds from undiscovered or unapplied species. For example, coral reefs are a key source of new marine natural products (NMNP) [114]. However, coral reefs are also forecasted to be a group negatively affected by ocean acidification [49]. While some reefs have been well-studied, others, particularly those that are mesophotic (low-light reefs occurring from 40–150 m depths), likely contain many species yet to be discovered. Consequently, these habitats remain potential sources of novel pharmaceuticals and NMNPs [115]. As ocean acidification is expected to negatively impact biodiversity, it will also impair our ability to prospect for new medications in such areas. Consequently, our current understanding of the true value of the existence of habitats, and the cost of their loss, is incomplete. We do recognize, however, that this inability to develop new medicines and other resources is an issue that will affect our ability to respond to all the issues considered here, from malnutrition and poisoning to respiratory issues and mental health impacts.

### 3.5. Interconnection of Pathways to Impacts

**Figure 1:**
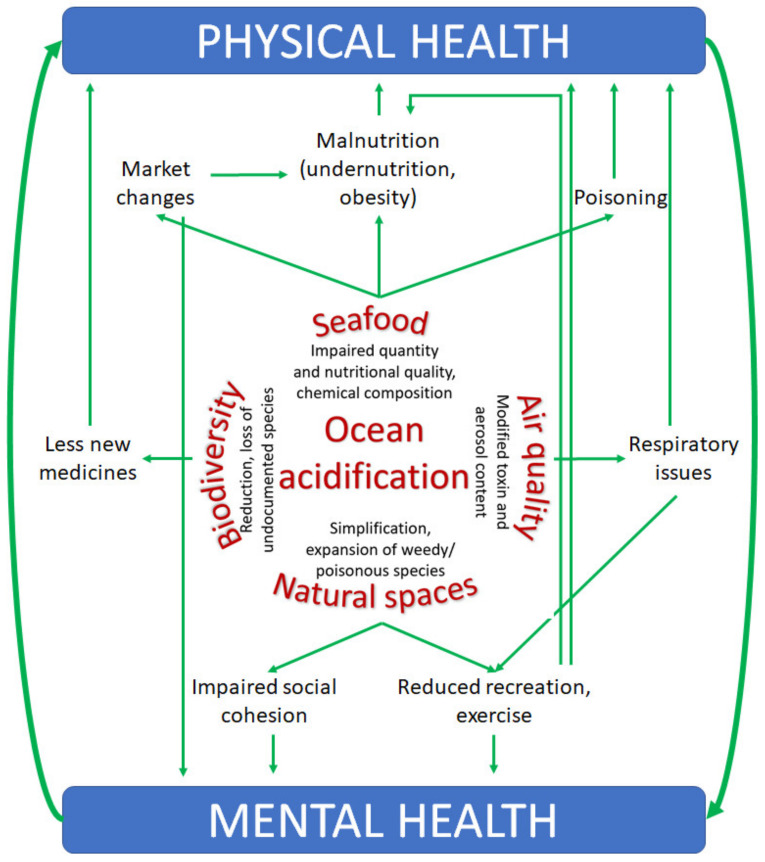
Ocean acidification is predicted to negatively affect four of the main services provided by the ocean (seafood, air quality, natural spaces, and marine biodiversity). All of these services are linked to physical and mental health through interconnected direct and indirect pathways.

As ocean acidification manifests, it is unlikely that people will experience any of its impacts singularly. Rather, the negative impacts of entrained ocean acidification on human health are set to be a product of the complex combinations of linkages (Figure 1). Moreover, it is important to note that physical and mental health and well-being do not occur in isolation, meaning it is useful to consider the combined influences of ocean acidification on overall well-being (Figure 1). For example, regions that experience high levels of toxins in seafood such that food resources are restricted may be more likely to also experience high levels of toxins in algal blooms such that air quality is impaired. Indeed, the toxins in algal blooms often propagate through trophic transfer and amplify in the seafoods consumed by humans. Therefore, the combination of these changes could lead to impacts on physical health in terms of potential poisoning and long-term malnutrition, as well as respiratory issues. Moreover, these experiences are likely to have negative mental health effects, which could compound the effects on physical health. Similarly, regions that experience losses in biodiversity will likely also experience a reduction in the quantity of seafood, driving malnutrition. While this malnutrition is likely to manifest as undernutrition, in some regions where alternative food sources are available, obesity could result due to substitution with less nutritious foods. The loss of biodiversity and habitat degradation will likely negatively impact natural spaces, reducing the incentive to experience these areas and exercise, potentially further reinforcing the trend toward obesity and the associated physical and mental health effects. A reduction in the amount of seafood available, either for wild-capture fisheries or aquaculture, would lead to changes in the market, with less available to sell. Such changes would impact physical health via malnutrition. Moreover, such shifts in the market would impair the job prospects of workers involved in these industries, producing negative mental health impacts. Again, it is anticipated these physical and mental health impacts would feed back and exacerbate each other’s effects.

## 4. Strategies to Enhance Benefits to, and Limit Negative Effects on, Human Health under Ocean Acidification

Limiting ocean acidification via implementation of CO_2_ emission mitigation strategies is the best way to maintain human health in this context. Key mitigation approaches could include those that combine measures aimed at the reduction of energy use by end-use sectors, decrease of net greenhouse gas emissions, decarbonization of energy supply, and carbon capture and sequestration through the enhancement of natural sinks or engineering techniques [116].

The effects of entrained ocean acidification can also be influenced by the adaptation solutions implemented [117]. While acidifying oceans drive changes in water chemistry that have flow-on negative effects in marine species and ecosystems, there is opportunity for managers to implement strategies to help coastal ecosystems adapt to these changes and, thus, optimize human health and well-being. Some of the approaches we outline may not only be effective in countering ocean acidification but also effective in countering other environmental changes, such as warming, pollution, and habitat degradation. Typically, these strategies involve: (a) conserving biodiversity and restoring and complex ecosystems, (b) monitoring and managing water quality at a local scale, and (c) adapting human activities and socio-ecological systems to ocean change.

### 4.1. Biodiversity Conservation and Restoration

While the changing climate brings many new challenges for biodiversity conservation, ocean acidification is among the key questions of importance in the context of retaining global biological diversity [32,118,119]. Management strategies that promote diversity, such as marine-protected areas (MPAs), restoration programs, or ecosystem-based management, could encourage resistance and resilience to acidification-related species loss [120,121,122]. Concern has, however, been raised that such approaches do not necessarily address population-level impacts [32]. While such strategies may be beneficial, potential barriers to implementation include insufficient funding, diverging interests, and the need for increased capacity [32]. Where approaches such as protected areas are implemented, their location and levels of protection will be critical to success; they will be most effective when situated to avoid hotspots of acidification or areas likely to protect genetic diversity or locally adapted populations [32,123,124].

### 4.2. Monitoring and Managing Water Quality at a Local Scale

Ocean acidification does not occur in isolation from other environmental changes but, rather, combines with locally driven changes. The development of centers of human populations has influenced the water quality of receiving marine ecosystems via outputs from industry and wastewater facilities, agricultural discharge, and urban runoff [125]. Such pollutants can affect the structure of the ecosystems and species we depend upon, with increasing evidence that these changes will be exacerbated by future global changes, such as ocean acidification [126]. Promisingly, it appears that where we effectively manage local-scale stressors, including water quality, the effects of global-scale changes can be reduced [127,128]. Consequently, a key part of managing change driven by global-scale stressors will be the effective monitoring of aquatic environments [125,129,130]. Such monitoring can be used to inform models that predict water quality, allowing a better understanding of human-water relations and identification of areas where we need to more effectively manage human-derived inputs.

### 4.3. Adapting Human Activities

Adapting to ocean changes will necessarily include efforts to adaptively manage socio–ecological systems [131]. Humans can adapt to ocean acidification to exploit beneficial opportunities or reduce damage (e.g., considering thriving species as a resource [132]). Hence, there are opportunities to adapt fisheries and aquaculture by iteratively changing management practices based on new experiences and insights.

Perhaps the first concrete example of a business responding to ocean acidification can be drawn from oyster aquaculture. In response to the increase in the area affected by pH upwelling around Oregon and Washington states (United States) driven by ocean acidification, a Willapa Bay-based oyster grower opened a hatchery in Hawaii to access an alternative source of larvae produced distant from coastal acidification [32,61]. While such actions benefit the businesses that source oysters from this hatchery, it is likely that rising costs of additional demand are now experienced in the Hawaiian system from which oyster larvae are being increasingly sourced. The capacity of businesses to undertake such responses will, however, not be consistent. That is, there are substantial investment costs associated with establishing an alternative source of larvae, which not all would be able to afford (e.g., those in developing nations).

There will be limits to our ability to adapt. That is, at a certain level of ocean acidification, ecosystems and lifecycles will be disrupted beyond a critical threshold. Where this occurs, and the world’s demand for secure sources of food continues to grow, boosting the human consumption of species that benefit from ocean acidification, such as fast-growing macroalgae, may help satisfy future demands [132]. Although marine food is not immune to overexploitation and needs to be managed sustainably [133], species that benefit from ocean acidification may recover more readily from harvesting than other species [134].

Altering consumption patterns can, however, have a range of outcomes depending on how the consumption pattern is altered. If consumption is directed toward food that has insufficient protein or nutrients in regions where alternatives to seafood are not available, malnourishment is likely to become more widespread in the local population. Such changes are particularly likely to be observed in developing nations and, especially, for poor coastal and island communities, since there would also be decreased livelihoods. Even where other alternatives are available, such as in developed countries, these are likely to be more processed and contain greater amounts of artificial colors, flavors, and preservatives. Consequently, such shifts toward these food sources could lead to obesity, increased cardiovascular disease, and other negative health effects in the long run [85].

### 4.4. The Socioeconomic Context of Affected Populations and Individuals—Environmental, Health, Economic, and Social Inequalities

It is also important to recognize, and account for, the socioeconomic context within which ocean acidification manifests and the implications that will have for the availability and management of resources critical to human health and well-being. For example, as fish are less abundant, this will likely lead to lower catches. The impacts of these lower catches are, however, going to be distributed unevenly across populations. That is, those who are able to afford the price of an increasingly rare resource will still have access, while those who are not will lose access to this resource. The management options available to cope with such losses are also likely to be limited in such populations. Such a pattern reflects a broader concern that climate change effects are currently, and will continue to be, stronger for populations at socioeconomic disadvantage, exacerbating the existing inequitable distribution of health and environmental challenges [135,136]. 

While we have focused here on the effects of ocean acidification on biota and their ecosystems, human health reflects much more than just these ecological systems. That is, the effects on human health and well-being, and the management strategies that will be most effective, are related to the interplay between individuals and institutions. The risks and costs to an individual and their broader community are mediated by human behaviors, social and government institutions, and physical infrastructures they provide [137]. As individuals, mediating effects pivot on sociodemographics (such as sex, age, race and ethnicity, marital and partner status, and education) and resourcing (such as housing, poverty status, and household and personal incomes). As communities, mediating effects will depend on community health and security, community attachment, social capital, and community resiliency. These individual-based and community-based influences interplay with each other and the adaptability and effectiveness of the institutions on which they rely.

## 5. Conclusions

Ocean acidification is anticipated to drive complex changes in the occurrence of individual species and ecological infrastructure from which human health and well-being benefit. Subsequent changes to human health and well-being can result from modifications to the food supply and food quality, respiratory issues, mental and physical health, and the treatment of diseases occurring due to acidification. To understand the scale and risks of such challenges, researchers may improve the value of their research by anticipating and monitoring for such changes. Yet, ocean acidification is intensifying in combination with climate change and other environmental stressors, necessitating the consideration of their combined effects. Moreover, we also need to consider trade-offs and feedbacks that occur with the range of relevant environmental conditions, as well as with changes in human behavior and demography (e.g., population movements and regional conflicts). We highlight approaches relevant when planning and adapting in this context, specifically mitigation and adaptation via biodiversity conservation, local management, and adapting human activities. We emphasize that the appropriateness and benefit to be obtained from these approaches will be dependent upon the socioeconomic context in which they are implemented. By recognizing these complexities, we may adapt our management of acidifying oceans so they not only reduce the harm but, also, enhance the benefits to human health and well-being.

## Figures and Tables

**Table 1:** Ocean acidification is predicted to negatively affect four of the main services provided by the ocean (seafood, air quality, natural spaces, and marine biodiversity). Here, we highlight key changes included in each pathway and relevant references.

Pathway of Ocean Acidification Impact	References
Pathway 1—malnutrition and poisoning via altered food quantity and quality	
• Reduced quantity	[30,31,32,33]
• Impaired nutritional composition	[27,34]
• Chemical contamination (pollutants)	[20,35,36,37]
• Redistribution and accumulation of natural toxins	[38,39]
Pathway 2—respiratory issues via impaired air quality	
• Enhanced aerosolization of natural toxins	[38,40]
Pathway 3—mental health impacts via modification of natural spaces	
• Loss of livelihoods	[41]
• Disruption of nature-based recreation, exercise, and connection	[42,43,44,45,46]
• Reduced social connections	[47,48]
Pathway 4—decreased opportunity to develop and obtain medical resources via loss of biodiversity	
• Loss of source of potential medical resources	[49,50]
